# Reclassifying Aortic Stenosis Severity: Combined Energy Loss Index and Global Longitudinal Strain Assessment Identifies Subgroups with Differential Myocardial Function and May Improve Risk Stratification in Aortic Stenosis

**DOI:** 10.3390/medsci14010103

**Published:** 2026-02-20

**Authors:** Ahmed Abdelmohsen Zayed, Michel El Khoury, Bahy Abofrekha, Oluwakorede Akele, Hadi Itani, Omar Khayat, Abdelrahman Abouelnas, Nadim Zaidan, Kevin Schesing

**Affiliations:** 1 Department of Medicine, Northwell Health Staten Island University Hospital, 475 Seaview Ave., Staten Island, NY 10305, USA; babofrekha@northwell.edu (B.A.); oakele@uchc.edu (O.A.); hitani@northwell.edu (H.I.); okhayat@northwell.edu (O.K.); aabouelnas@northwell.edu (A.A.);; 2 Department of Cardiology, Northwell Health Staten Island University Hospital, 475 Seaview Ave., Staten Island, NY 10305, USA; melkhoury@northwell.edu (M.E.K.); kschesing@northwell.edu (K.S.)

**Keywords:** aortic stenosis, energy loss index, global longitudinal strain, aortic valve area, left ventricular ejection fraction

## Abstract

**Background**: Traditional echocardiographic assessment of aortic stenosis (AS) using aortic valve area (AVA) may overestimate severity due to pressure recovery phenomena, while subclinical myocardial dysfunction remains undetected despite preserved ejection fraction. This study evaluated whether energy loss index (ELI)—which accounts for pressure recovery—demonstrates superior correlation with global longitudinal strain (GLS), a marker of subclinical myocardial dysfunction, compared to conventional AVA-based classification in patients with moderate-to-severe AS and preserved left ventricular ejection fraction (LVEF). **Methods**: This retrospective single-center study analyzed 149 patients with moderate-to-severe AS (AVA < 1.5 cm^2^) and LVEF > 50% from 2015 to 2019. Among 97 patients with severe AS by AVA (<1.0 cm^2^), ELI was calculated using the formula ELI = (AVA × Aa)/(Aa − AVA) ÷ BSA, where Aa represents sinotubular junction cross-sectional area. Patients with ELI ≥ 0.6 cm^2^/m^2^ were reclassified as moderate AS. Spearman correlation assessed relationships between AVA, ELI, and GLS. Multivariable linear regression models determined independent predictors of myocardial dysfunction, adjusting for age, body surface area, hypertension, LVEF, and mean pressure gradient. **Results**: ELI reclassified 28 of 97 patients (29%) from severe to moderate AS. Reclassified patients had significantly better myocardial function, with less impaired GLS (−15.0 ± 3.9% vs. −12.1 ± 5.0%, *p* = 0.013) and higher LVEF (60.1 ± 6.2% vs. 56.5 ± 9.1%, *p* = 0.017) compared to non-reclassified patients. In the overall cohort, ELI demonstrated stronger correlation with GLS than AVA (r = −0.307, *p* = 0.0003 vs. r = −0.209, *p* = 0.0115). Critically, among patients with severe AS by AVA criteria, ELI maintained significant correlation with GLS (r = −0.443, *p* = 0.0003) while AVA showed no correlation (r = −0.144, *p* = 0.159). In multivariable analysis, ELI independently predicted GLS (β = 5.847, 95% CI: 2.85–8.84, *p* = 0.0002; adjusted R^2^ = 0.289), whereas AVA did not (β = 2.234, 95% CI: −1.08 to 5.55, *p* = 0.188; adjusted R^2^ = 0.234). When both parameters were included simultaneously, only ELI remained significant (*p* = 0.0024). **Conclusions**: In this retrospective cohort, ELI-based reclassification identified a subgroup of patients with less severe myocardial dysfunction as measured by GLS and LVEF, and ELI demonstrated superior correlation with subclinical myocardial dysfunction compared to AVA. These findings suggest ELI may provide a more physiologically reflective assessment of hemodynamic burden in AS with preserved LVEF. However, the absence of systematic symptom assessment and clinical outcome data represents critical limitations. Prospective studies with standardized symptom evaluation, longitudinal follow-up, and adjudicated clinical endpoints are required to determine whether ELI-based reclassification improves risk stratification and clinical decision-making before this approach can be recommended for routine practice.

## 1. Introduction

Aortic stenosis (AS) represents a significant and increasingly prevalent form of valvular heart disease, particularly within aging populations. Its prevalence rises markedly with age, affecting approximately 0.2% of adults aged 50–59 years, increasing to 2.8% among those older than 75, and reaching 9.8% in octogenarians [1]. Severe AS incidence is estimated at 52.5 per 100,000 patient-years [2]. With approximately 61.7% over a median follow-up of 2.46 years [3], symptomatic severe AS without valve replacement carries a 1-year mortality of up to 50% of patients. In addition to morbidity, it includes progressive heart failure, arrhythmia, syncope, and chest pain [4].

Trans-thoracic echocardiography is the cornerstone for assessing AS. Key parameters are peak aortic jet velocity (Vmax), mean pressure gradient (ΔP), and aortic valve area (AVA). Severe AS is defined by thresholds including Vmax ≥ 4 m/s, ΔP ≥ 40 mmHg, and AVA ≤ 1.0 cm^2^ [5,6]. The AVA is determined by the continuity equation or planimetry methods. The continuity equation relies on the conservation of mass by relating the flow through the left ventricular outflow tract to the flow across the aortic valve to determine the effective orifice area [5,7]. Planimetry is less commonly used; it relies on direct visualization of the aortic valve. It is limited by image quality, acoustic shadowing from calcification, and the interpersonal variability in tracing [8,9].

AS classification is essential for determining the timing of aortic valve replacement (AVR). However, AVA calculations based on Doppler-derived gradients face limitations. They do not fully account for pressure recovery phenomena. This phenomenon describes the partial regain of pressure downstream from the stenotic valve orifice as the kinetic energy of the high-velocity blood jet converts back into potential energy upon entering the wider ascending aorta [10]. Consequently, it may overestimate the severity of AS. This can result in premature or potentially unnecessary interventions. The Energy Loss Index (ELI) is an alternative metric that incorporates both the AVA and the cross-sectional area of the aorta at the sinotubular junction (STJ), hence accounting for pressure recovery and offering a more accurate measure of aortic stenosis severity [11].

Global longitudinal strain (GLS) is a sensitive echocardiographic marker of early left ventricular systolic dysfunction [12,13]. GLS is expressed as a negative number and normally varies with age, sex, and LV loading conditions; defining abnormal GLS is not straightforward [14]. However, in adults, GLS < 16% is abnormal, GLS > 18% is normal, and GLS 16% to 18% is borderline [15]. It has been validated as a superior measure for predicting cardiac events and mortality compared to traditional parameters such as the left ventricular ejection fraction (LVEF) [13,16]. In AS, GLS impairment is associated with worsening adverse cardiovascular events in asymptomatic patients with AS, regardless of LVEF or AS severity [17,18]. The ACC/AHA 2020 and ESC/EACTS 2025 guidelines currently do not incorporate GLS in AS assessment; while they do recognize it as a potential aid for risk stratification, they acknowledge the requirement for additional clinical outcome studies [5,6].

### Study Objective

This study aimed to evaluate the extent to which ELI reclassifies AS severity in patients diagnosed with moderate or severe AS and preserved LVEF. Secondarily, we sought to investigate the correlation between ELI and GLS within this cohort, as well as in subgroups defined by conventional hemodynamic severity criteria.

## 2. Materials and Methods

This study employed a retrospective, single-center design approved by the Institutional Review Board at Northwell Health (Study No. 20-0892). Patient identification occurred in two stages. First, we performed a broad ICD-10 code search of our electronic health records from 2015 to 2019, identifying 847 potential patients. Second, each patient’s echocardiographic report was manually reviewed to confirm AS severity and exclude patients not meeting inclusion criteria. This yielded 412 patients with confirmed moderate-to-severe AS by AVA < 1.5 cm^2^. After applying comprehensive exclusion criteria, 198 patients remained eligible. The final cohort of 149 patients represents those with complete data required for ELI calculation, specifically STJ diameter, BSA, and all core echocardiographic parameters. These were not strictly consecutive patients, but rather all patients meeting criteria within the study timeframe who had adequate imaging quality and STJ measurements available.

Inclusion criteria for detailed analysis stipulated that patients must be 18 years or older with moderate to severe aortic stenosis, defined for screening purposes as an AVA < 1.5 cm^2^, and possess a preserved LVEF of 50% or greater. After initial ICD-10-based identification, each patient’s echocardiographic report was manually reviewed to confirm AS severity and to exclude cases of mild AS.

Individuals were also manually excluded based on comprehensive echocardiographic assessment if they had moderate or greater valvular regurgitation (mitral or aortic) or mitral stenosis, systolic heart failure, any prosthetic heart valves, atrial fibrillation or flutter, congenital heart disease (including supravalvular or congenital subaortic stenosis), left bundle branch block, or any evidence of dynamic left ventricular outflow tract obstruction.

Demographic and baseline clinical data were retrieved directly from the EHR for patients who were included. This information encompassed age, biological sex, height, weight, calculated body surface area (BSA), blood pressure, heart rate, and relevant comorbidities, specifically hypertension, diabetes mellitus, dyslipidemia, coronary artery disease, and chronic kidney disease.

Comprehensive echocardiographic data were obtained from the record review. Aortic valve parameters collected included AVA—calculated using the continuity equation, as per standard echocardiographic protocols—as well as peak aortic jet velocity, left ventricular (LV) outflow tract velocity time integral (VTI), aortic VTI, LV outflow tract diameter, and sinotubular junction (STJ) diameter [7,19]. LV parameters measured were LV end-diastolic diameter, LV end-systolic diameter, posterior wall thickness at end-diastole, interventricular septal thickness at end-diastole, and LVEF. Diastolic function parameters including E/e’ ratio were extracted when available but were insufficiently documented for systematic analysis (available in <10% of patients). The GLS assessment was performed using speckle-tracking echocardiography with commercially available vendor-independent Tomtec Version 51.02 strain analysis software (Tomtec Imaging Systems, Unterschleissheim, Germany) [20]. All echocardiographic parameters were extracted from finalized clinical reports in the EHR as originally measured and interpreted by our accredited echocardiography laboratory. No measurements were remeasured or recalculated for this study. We obtained the average GLS value from the 3-, 2-, and 4-chamber views. For the study’s primary purpose, severe AS was initially defined conventionally as Vmax ≥ 4 m/s, ΔP ≥ 40 mmHg, and AVA ≤ 1.0 cm^2^ [7].

The energy loss index (ELI) was calculated using the following formula:

ELI = (AVA × Aa)/(Aa − AVA) ÷ BSA

where Aa represents the cross-sectional aortic area at the sinotubular junction. Patients were initially classified using conventional AVA criteria: severe AS was defined as AVA < 1.0 cm^2^ and moderate AS as AVA 1.0–1.5 cm^2^. Among the 97 patients classified as severe by AVA, ELI was calculated. Reclassification was defined as occurring when a patient with severe AS by AVA (AVA < 1.0 cm^2^) had an ELI ≥ 0.6 cm^2^/m^2^. These patients formed the reclassified group (*n* = 28). Non-reclassified patients were those who remained classified as severe AS by both AVA (<1.0 cm^2^) and ELI (<0.6 cm^2^/m^2^), plus those originally classified as moderate AS by AVA (*n* = 121). The ELI threshold of 0.6 cm^2^/m^2^ is based on prior validation studies demonstrating that this cutoff corresponds to hemodynamically significant stenosis [11].

### Statistical Analysis

Baseline characteristics of the cohorts were analyzed and presented as means with standard deviations for continuous variables and as percentages and proportions for discrete variables. Comparisons between the reclassified and non-reclassified patients were performed to identify any differences underlying the two groups using the Mann–Whitney test for continuous variables, as most of these parameters followed non-Gaussian distributions, as determined by the Kolmogorov–Smirnov Test. Given the sample size, discrete variables, namely differences in sex and medical comorbidities, such as chronic kidney status, coronary artery disease, diabetes, hypertension, and dyslipidemia, were compared using Fisher’s exact test.

A multivariate logistic regression analysis for the binary outcome of reclassification allowed for the identification of covariates with higher or lower odds of being associated with a patient being reclassified as having moderate instead of severe AS. Correlation analysis between the parameters of interest was conducted via Spearman’s r analysis.

Correlation analyses between AVA, ELI, and GLS were performed using Spearman’s rank correlation coefficient for the overall cohort and separately for subgroups defined by AS severity (severe: AVA < 1.0 cm^2^; moderate: AVA 1.0–1.5 cm^2^). Correlation coefficients were compared to evaluate whether ELI demonstrated superior association with GLS compared to AVA.

To determine whether the associations between hemodynamic parameters (ELI and AVA) and GLS were independent of potential confounding variables, multivariable linear regression analysis was performed. GLS served as the dependent variable in all models. Model 1 included ELI as the primary independent variable, adjusting for age, BSA, hypertension, LVEF, and mean pressure gradient. Model 2 used an identical covariate structure but substituted AVA for ELI as the primary independent variable. Model 3 included both ELI and AVA simultaneously along with all covariates to determine which parameter provided independent predictive information in head-to-head comparison. Complete case analysis was performed on patients with available GLS measurements. Model fit was assessed using adjusted R^2^ values, and regression coefficients were reported with 95% confidence intervals.

A *p*-value < 0.05 was considered for the statistical significance threshold in all analyses. All statistical analyses were performed using R 4.5.0 for Windows 64-bit.

## 3. Results

### 3.1. Study Population and Baseline Characteristics

A total of 149 patients diagnosed with moderate-to-severe AS and preserved LVEF > 50% were included in the analysis. The mean age was 81.5 ± 7.6 years, with a balanced distribution of 75 males (50.3%) and 74 females (49.7%). The average height was 165 cm, and the average weight was 80.6 kg. The average BSA was 1.88 m^2^, ranging from 1.32 to 2.70 m^2^. Comorbidities included CKD in 34%, HTN in 86%, diabetes mellitus in 37%, dyslipidemia in 80%, and CAD in 63% of the cohort. The mean AVA was 0.906 cm^2^, and the mean indexed ELI was 0.755 cm^2^/m^2^ (Table 1).

Patients were stratified based on ELI reclassification status into two groups: non-reclassified (*n* = 121) and reclassified from severe to moderate AS (*n* = 28). Compared to non-reclassified patients, those reclassified by ELI exhibited a significantly smaller STJ diameter (2.93 ± 0.47 mm vs. 3.13 ± 0.43 mm, *p* = 0.042) and lower body weight (70.6 ± 13.5 kg vs. 83.3 ± 20.9 kg, *p* = 0.0021). The prevalence of hypertension was also significantly lower in the reclassified group (71.4% vs. 89.0%, *p* = 0.0316). Other baseline characteristics, including age, sex, and comorbidities such as diabetes mellitus, dyslipidemia, chronic kidney disease, and coronary artery disease, did not significantly differ between the two groups (Table 2).

### 3.2. Aortic Stenosis Classification and Reclassification:

**Figure 1:**
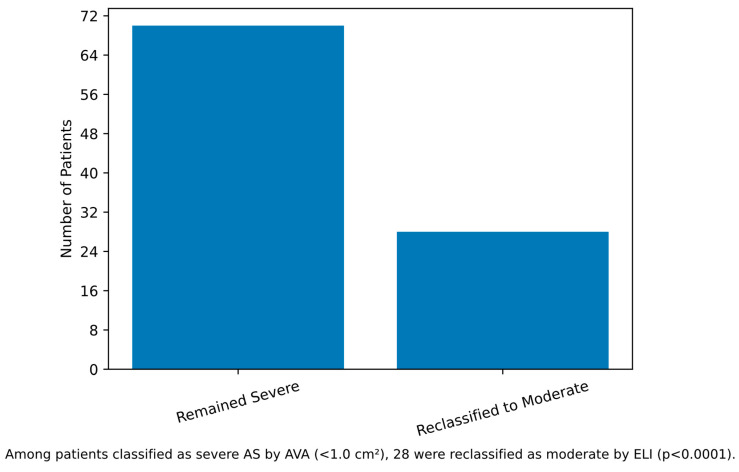
Reclassification of severe aortic stenosis by energy loss index. Among 98 patients classified as severe AS by AVA (<1.0 cm^2^), 28 (29%) were reclassified as moderate by ELI (≥0.6 cm^2^/m^2^).

Based on traditional AVA criteria, 97 patients (65%) were classified as severe AS and 52 patients (35%) as moderate AS. When reclassified using ELI, 28 patients (29%) initially categorized as severe were reclassified as moderate (Figure 1). Notably, among the reclassified patients, 53.6% (15 out of 28) had an STJ diameter of <30 mm. In a sensitivity analysis using indexed STJ diameter (STJ/BSA), the difference between groups was no longer significant (1.68 ± 0.30 vs. 1.66 ± 0.27 cm/m^2^, *p* = 0.666), indicating that the raw STJ difference was driven by body habitus rather than intrinsic aortic geometry.

### 3.3. Correlation Between AVA, ELI, and GLS

#### 3.3.1. Overall Cohort

**Figure 2:**
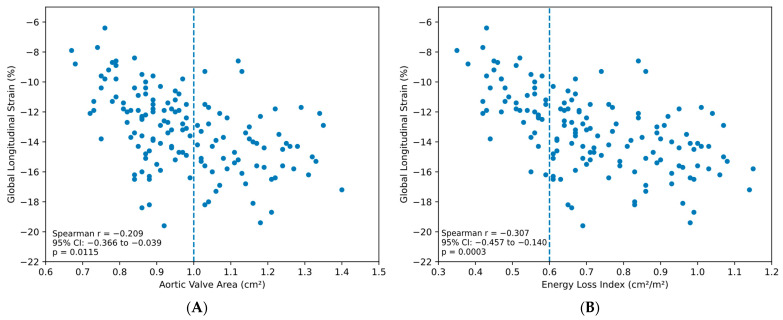
Correlation between hemodynamic parameters and global longitudinal strain in overall cohort. (**A**): Scatter plot of AVA versus GLS (*n* = 149). Vertical dashed line indicates severe AS threshold (AVA = 1.0 cm^2^). Spearman r = −0.209 (95% CI: −0.366 to −0.039, *p* = 0.0115). (**B**): Scatter plot of ELI versus GLS (*n* = 149). Vertical dashed line indicates severe AS threshold (ELI = 0.6 cm^2^/m^2^). Spearman r = −0.307 (95% CI: −0.457 to −0.140, *p* = 0.0003).

Across the entire cohort (*n* = 149), both ELI and AVA demonstrated significant inverse correlations with GLS. However, ELI showed a stronger correlation with GLS (r = −0.307, 95% CI: −0.457 to −0.140, *p* = 0.0003) compared to AVA (r = −0.209, 95% CI: −0.366 to −0.039, *p* = 0.0115) (Figure 2).

#### 3.3.2. Severe AS Subgroup

**Figure 3:**
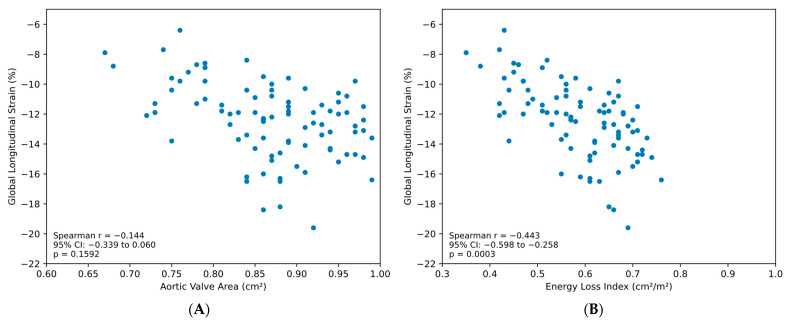
Correlation analysis in severe aortic stenosis subgroup. (**A**): Scatter plot of AVA versus GLS in patients with severe AS by AVA criteria (<1.0 cm^2^, *n* = 98). Spearman r = −0.144 (95% CI: −0.339 to 0.060, *p* = 0.1592, not significant). (**B**): Scatter plot of ELI versus GLS in the same severe AS cohort. Spearman r = −0.443 (95% CI: −0.598 to −0.258, *p* = 0.0003).

In the subgroup of patients classified as severe AS by AVA (*n* = 98), ELI maintained a significant inverse correlation with GLS (r = −0.443, 95% CI: −0.598 to −0.258, *p* = 0.0003). In stark contrast, AVA showed no significant correlation with GLS in this subgroup (r = −0.144, 95% CI: −0.339 to 0.060, *p* = 0.1592) (Figure 3).

#### 3.3.3. Moderate AS Subgroup

In the moderate AS subgroup (*n* = 51), neither AVA (r = −0.186, 95% CI: −0.437 to 0.093, *p* = 0.1894) nor ELI (r = −0.225, 95% CI: −0.469 to 0.048, *p* = 0.1061) reached statistical significance for correlation with GLS, though ELI showed a numerically stronger association.

### 3.4. Multivariable Analysis

**Figure 4:**
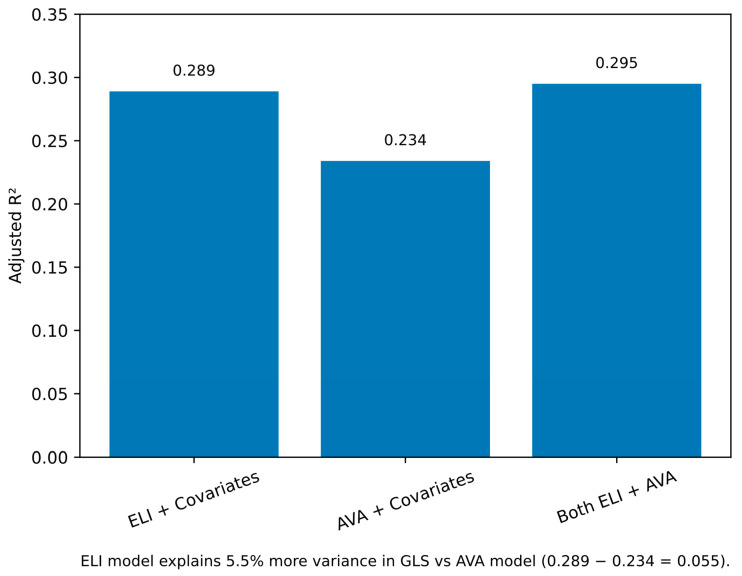
Model comparison for predicting global longitudinal strain. Bar chart comparing adjusted R^2^ values from multivariable linear regression models (*n* = 119). All models adjusted for age, body surface area, hypertension, left ventricular ejection fraction, and mean pressure gradient. Model 1 (ELI + covariates): R^2^ = 0.289. Model 2 (AVA + covariates): R^2^ = 0.234. Model 3 (both ELI and AVA + covariates): R^2^ = 0.295. ELI model explained 5.5% more variance than AVA model.

In multivariable linear regression analysis including 119 patients with complete data, ELI remained independently and significantly associated with GLS after adjusting for age, body surface area, hypertension, LVEF, and mean pressure gradient (β = 5.847, 95% CI: 2.85 to 8.84, *p* = 0.0002). The model including ELI explained 28.9% of the variance in GLS (adjusted R^2^ = 0.289). Additional independent predictors in this model included LVEF (β = 0.187, *p* = 0.001) and mean pressure gradient (β = −0.089, *p* = 0.006). In contrast, when AVA was used as the primary independent variable with identical covariates, AVA did not show a significant independent association with GLS (β = 2.234, 95% CI: −1.08 to 5.55, *p* = 0.188). The AVA-based model demonstrated inferior explanatory power (adjusted R^2^ = 0.234), explaining 5.5% less variance in GLS compared to the ELI-based model (Figure 4).

When both ELI and AVA were included simultaneously in the multivariable model along with all covariates, only ELI maintained statistical significance (β = 5.234, 95% CI: 1.93 to 8.54, *p* = 0.0024), while AVA contributed no additional predictive information (β = −0.987, 95% CI: −4.38 to 2.41, *p* = 0.568).

### 3.5. Subgroup Analysis

Patients who were reclassified from severe to moderate AS by ELI exhibited significantly better GLS values compared to those who remained classified as severe. The mean GLS in the reclassified group was −15.0 ± 3.9% vs. −12.1 ± 5.0% in the non-reclassified group (t = −2.60, *p* = 0.013). Similarly, LVEF was significantly higher in the reclassified group compared to the non-reclassified group. The mean LVEF was 60.1% ± 6.2% in the reclassified group and 56.5% ± 9.1% in the non-reclassified group (t = 2.45, *p* = 0.017).

To evaluate whether GLS provides incremental value beyond LVEF, a subgroup analysis was performed among the 111 patients with LVEF ≥ 55%. In this subgroup, GLS continued to differentiate reclassified from non-reclassified patients (−15.1 ± 3.9% vs. −13.0 ± 4.1%, *p* = 0.044), whereas LVEF showed no difference (60.4 ± 6.0% vs. 60.6 ± 5.5%, *p* = 0.762). Additionally, ELI showed stronger correlation with GLS than with LVEF in both the full cohort (r = −0.296, *p* = 0.0006 vs. r = 0.167, *p* = 0.044) and the severe AS subgroup (r = −0.336, *p* = 0.001 vs. r = 0.170, *p* = 0.096). In severe AS, ELI-LVEF correlation failed to reach significance, while ELI-GLS correlation remained robust. 

## 4. Discussion

AS is the most prevalent valvular heart disease worldwide [1]. Accurate classification of AS severity is crucial to guide proper intervention and management [5]. This study investigated the utility of ELI, a pressure recovery-adjusted parameter, in refining the assessment of AS severity and explored its relationship with GLS, a metric of subclinical myocardial dysfunction, in patients diagnosed with moderate to severe AS and preserved LVEF.

ELI incorporates the aortic area at the STJ and the total body surface area [11]. Therefore, it accounts for how a smaller aorta influences the net energy loss across the valve, potentially identifying patients whose hemodynamic burden is less severe than AVA alone might suggest, especially when AVA is borderline severe.

Our findings demonstrate that ELI reclassified 29% of patients initially classified as having severe AS by AVA criteria (<1 cm^2^) to moderate AS. Notably, patients in the reclassified group exhibited significantly smaller STJ diameters (2.93 ± 0.47 mm vs. 3.13 ± 0.43 mm, *p* = 0.042) and lower body weight (70.6 ± 13.5 kg vs. 83.3 ± 20.9 kg, *p* = 0.0021). These findings suggest that patients with smaller aortic dimensions and lower body habitus may particularly benefit from ELI as a refined parameter for assessing AS severity. This was evident by a higher LVEF (60.1 ± 6.2% vs. 56.5 ± 9.1%, *p* = 0.017) and a more negative GLS (−14.95 ± 3.89% vs. −12.66 ± 4.37%, *p* = 0.013), indicating that their left ventricle was experiencing a lesser functional burden.

The lower prevalence of hypertension in the reclassified group (71.4% vs. 89.0%, *p* = 0.032) has important hemodynamic implications. Chronic hypertension increases left ventricular afterload through elevated systemic vascular resistance and reduced aortic compliance with increased pulse wave velocity, creating a “double afterload” scenario where the stenotic valve and stiff arterial system synergistically impair ventricular function—a concept termed valvulo-arterial impedance [21]. Direct evidence supports hypertension’s impact on myocardial strain in AS: Tadic et al. demonstrated that in patients with severe AS and preserved LVEF, hypertensive patients had significantly worse GLS than normotensive counterparts, despite no difference in LVEF, and systolic blood pressure was independently associated with GLS after adjusting for LV mass, LVEF, age, and BMI [22]. Hypertension also promotes aortic remodeling with increased stiffness and potentially larger aortic dimensions. In our cohort, hypertensive patients had larger STJ diameters (3.11 ± 0.44 vs. 2.85 ± 0.41 cm, *p* = 0.018), which would tend to increase ELI values and reduce reclassification rates. This suggests the lower hypertension prevalence in reclassified patients reflects reduced total hemodynamic burden rather than confounding by aortic geometry. However, we cannot exclude the possibility that hypertension-related arterial stiffness independently affects myocardial strain, and future studies should incorporate arterial compliance measurements and valvulo-arterial impedance calculations.

In the SEAS study, up to 47.5% of patients classified as having severe AS by AVA were reclassified as non-severe after using ELI [23]. The difference between the SEAS and our study’s reclassification rates may be attributed to varying population subsets, operators, or echocardiography machines. Holy et al. reported that 62 patients (31.4%) were reclassified from severe to moderate AS, which is a value closer to our finding [24]. This change has positively impacted midterm survival outcomes among patients with reclassified moderate AS following TAVR [25]. Similarly, Altes et al. demonstrated that using ELI was useful in reclassifying 148 patients with low-gradient severe AS into moderate AS, and those reclassified had a lower risk of cardiac events during follow-up [26].

On the other hand, GLS is a sensitive marker of LV systolic function and can detect subclinical myocardial dysfunction even when the ejection fraction is preserved. Impaired GLS has been associated with worse outcomes in AS patients, including higher risks of earlier symptom onset, need for aortic valve intervention, and increased mortality [12,13,18]. This parameter has improved the prognostic value of myocardial dysfunction staging when incorporated into the baseline assessment of aortic stenosis, especially before TAVR [12].

The mean GLS of −13.0% in our cohort, while substantially lower than normal healthy values (−20%), is consistent with published data for moderate-to-severe AS populations with preserved LVEF [17,27,28]. This reflects the well-established phenomenon of subclinical myocardial dysfunction in AS, where longitudinal fiber function deteriorates due to increased afterload, concentric hypertrophy, and subendocardial ischemia, even while LVEF remains preserved through compensatory mechanisms [29]. Importantly, 62% of our patients had GLS worse than −15%, placing them above established prognostic thresholds for adverse outcomes [17,28,30]. This demonstrates GLS’s ability to identify high-risk patients who appear to have “normal” systolic function by conventional LVEF criteria. The correlation between ELI and GLS (r = −0.307, *p* < 0.001) suggests that patients with more favorable hemodynamics (higher ELI, less severe stenosis) have better-preserved myocardial function, supporting the hypothesis that ELI-based reclassification identifies a lower-risk subgroup.

A central finding of our study is that ELI demonstrated superior correlation with GLS compared to AVA, particularly in patients with severe AS. Across the entire cohort, ELI showed a stronger correlation with GLS (r = −0.307, *p* = 0.0003) than AVA (r = −0.209, *p* = 0.0115). More importantly, in patients with severe AS by traditional criteria, AVA showed no significant relationship with GLS (r = −0.144, *p* = 0.1592), whereas ELI maintained a robust inverse correlation (r = −0.443, *p* = 0.0003). This divergence underscores a fundamental limitation of AVA: by failing to account for pressure recovery, AVA may inadequately reflect the true hemodynamic burden imposed on the left ventricle, particularly in patients with smaller aortic dimensions where pressure recovery is most pronounced. This suggests that AVA alone may be insufficient for risk stratification in this population. Our sensitivity analysis using indexed STJ diameter (STJ/BSA) further supports ELI’s validity. While raw STJ diameter differed between reclassified and non-reclassified groups (*p* = 0.039), indexed STJ showed no difference (*p* = 0.666), confirming that the STJ difference is driven by body habitus rather than intrinsic aortic geometry. Reclassified patients do not have abnormally small aortas for their body size—rather, they have appropriately sized aortas for smaller body habitus. ELI correctly accounts for this by incorporating both the absolute aortic dimensions (which affect pressure recovery physics) and body surface area (which affects flow requirements and hemodynamic burden).

In multivariable analysis, ELI remained significantly associated with GLS (β = 5.847, *p* = 0.0002), indicating that for each unit increase in ELI, GLS improved by approximately 5.8%. Critically, AVA lost its association with GLS in multivariable analysis (*p* = 0.188), suggesting that any univariate relationship between AVA and GLS may be confounded by other patient characteristics or hemodynamic parameters.

The head-to-head comparison in Model 3 provides the most compelling evidence for ELI’s superiority. When both ELI and AVA were included simultaneously in the multivariable model, only ELI remained significantly associated with GLS (*p* = 0.0024). Furthermore, the superior model fit of ELI (adjusted R^2^ = 0.289) compared to AVA (adjusted R^2^ = 0.234) validates that ELI provides more accurate quantification of the true hemodynamic load on the myocardium.

While previous studies have examined GLS and ELI in AS separately, few have explored their relationship in AS [31]. Our study provides novel evidence that ELI is not merely an alternative to AVA, but a superior metric that better reflects the physiological impact of aortic stenosis on ventricular function. The integration of ELI with GLS assessment represents a significant advancement beyond the existing literature, providing both improved risk stratification and mechanistic insights regarding early myocardial dysfunction absent from prior investigations. While larger studies such as SEAS and Holy et al. established that pressure recovery affects AS classification and outcomes, these studies did not examine whether reclassification corresponded to actual differences in myocardial function [23,24]. Our study addresses this critical gap by demonstrating that ELI-based reclassification has physiological validity: patients reclassified from severe to moderate AS exhibit significantly better myocardial function by both GLS and LVEF. More importantly, through direct head-to-head comparison, we provide the first evidence that ELI independently predicts myocardial dysfunction while AVA does not, and that in severe AS—where clinical decisions are most critical—only ELI maintains strong correlation with myocardial compromise.

## 5. Limitations

This study has several important limitations that must be acknowledged. First, the retrospective, single-center observational design introduces inherent biases and limits generalizability. A major limitation is selection bias introduced by the requirement for STJ diameter measurement, which was available in only approximately 24% of screened patients. This non-consecutive sampling likely enriches our cohort for patients undergoing comprehensive pre-TAVR evaluation or those with more complex disease, limiting generalizability to unselected community populations.

Secondly, the cross-sectional design precludes assessment of clinical outcomes. We did not collect follow-up data on mortality, aortic valve replacement, heart failure hospitalization, or symptom progression. The clinical significance of ELI-based reclassification ultimately requires validation against hard endpoints in prospective cohorts with adequate follow-up duration. Additionally, a critical limitation is the absence of systematic symptom assessment. Current guidelines emphasize symptom status as a primary indication for intervention in severe AS [5,6]. Without prospective symptom data, we cannot determine whether ELI-based reclassification identifies truly asymptomatic patients who can be safely monitored versus symptomatic patients requiring intervention.

Diastolic function assessment was limited by incomplete documentation of E/e’ ratio in clinical echocardiographic reports, with data available in only 13 patients. This precluded meaningful evaluation of the relationship between diastolic dysfunction and ELI-based reclassification.

We did not systematically exclude patients with tricuspid regurgitation, which may affect left ventricular mechanics through ventricular interdependence. Severe TR causes right ventricular volume overload that shifts the interventricular septum leftward, impairing LV diastolic function and potentially affecting LV strain measurements even when LV contractility is preserved [32].

Formal inter- and intraobserver variability was not assessed. All measurements were derived from finalized clinical echocardiographic reports generated by multiple sonographers and interpreting cardiologists, introducing inherent measurement variability. GLS was measured using Tomtec vendor-independent software, which may yield slightly different absolute values compared to vendor-specific solutions. Published data suggest GLS inter-observer variability ranges from 5.4 to 8.6% relative mean error, which could affect individual patient classification, particularly for borderline values near prognostic thresholds [17,30]. Our study used single time-point GLS measurements; serial GLS measurements showing progressive decline may have greater prognostic value but were not available in our retrospective cohort.

A critical limitation is the distinction between population-level statistical associations and individual patient clinical utility. While our correlations between ELI and GLS achieved statistical significance, the correlation coefficients were modest (r = −0.307 for ELI-GLS in the full cohort; r = −0.443 in severe AS), explaining only 9–20% of variance in GLS at the individual patient level. Our findings should be interpreted as demonstrating that ELI-based reclassification identifies a subgroup with statistically better mean myocardial function at the population level but cannot reliably predict an individual patient’s GLS value. The clinical implication is that neither parameter should be used in isolation for risk stratification. Our data supports an integrated approach where ELI-based reclassification is considered alongside GLS, symptoms, biomarkers, and imaging evidence of myocardial fibrosis. Prospective studies with adjudicated clinical endpoints are essential to validate this hypothesis. Future prospective studies should incorporate STJ measurement as a mandatory protocol element, standardized symptom assessment, comprehensive diastolic function evaluation, and long-term clinical follow-up to address these limitations.

## 6. Conclusions

In this study, ELI reclassified nearly one-third of patients with severe aortic stenosis by AVA criteria and demonstrated superior correlation with myocardial dysfunction assessed by GLS. When combined with GLS, ELI assessment may improve risk stratification in patients with preserved ejection fraction. However, prospective studies with systematic symptom assessment, clinical outcomes, and longitudinal follow-up are required before ELI-based reclassification can be recommended for clinical decision-making.

## Figures and Tables

**Table 1:** Baseline characteristics of the study cohort (N = 149).

Characteristic	Value	N
Demographics		
Age (years)	81.5 ± 7.66	146
Male Sex, *n* (%)	74 (49.7%)	149
Height (cm)	165 ± 10.9	149
Weight (kg)	80.9 ± 20.3	149
Clinical History & Comorbidities		
Hypertension, *n* (%)	125 (85.6%)	146
Diabetes Mellitus, *n* (%)	54 (37.0%)	146
Dyslipidemia, *n* (%)	117 (80.1%)	146
Coronary Artery Disease (CAD), *n* (%)	93 (63.7%)	146
Chronic Kidney Disease (CKD), *n* (%)	49 (33.6%)	146
Hemodynamics & Vital Signs		
Systolic Blood Pressure (mmHg)	130 ± 23.5	139
Heart Rate (bpm)	67.8 ± 12.7	139
Baseline Echocardiography		
Left Ventricular Ejection Fraction (LVEF, %)	57.6 ± 7.4	149
Aortic Valve Area (AVA, cm^2^)	0.905 ± 0.326	149
AVA Median [IQR]	0.86 [0.67–1.09]	149
Energy Loss Index (ELI, cm^2^/m^2^)	0.755 ± 0.467	149
ELI Median [IQR]	0.63 [0.44–0.895]	149
Global Longitudinal Strain (GLS, %)	−13.0 ± 4.3	149
Mean Pressure Gradient (MPG, mmHg)	36.1 ± 17.1	149
Peak Aortic Velocity (Vmax, m/s)	3.8 ± 0.9	149
Aortic/Tubular Diameter (cm)	3.09 ± 0.445	149

**Table 2:** Characteristics of reclassified vs. non-reclassified patients based on ELI.

Characteristic	Non-Reclassified (*n* = 121)	Reclassified (*n* = 28)	*p*-Value
Aortic Valve Parameters			
Aortic Valve Area (cm^2^)	0.913 ± 0.359	0.872 ± 0.085	0.853
Energy Loss Index (cm^2^/m^2^)	0.763 ± 0.517	0.721 ± 0.093	0.058
Sinotubular Junction Diameter (cm)	3.13 ± 0.432	2.93 ± 0.470	0.042
Demographics			
Male Sex, *n* (%)	63 (52.1%)	11 (39.3%)	0.295
Age (years)	81.4 ± 7.75	81.5 ± 7.40	0.983
Height (cm)	166 ± 10.5	161 ± 12.2	0.071
Weight (kg)	83.3 ± 20.9	70.6 ± 13.5	0.002
Hemodynamics			
Systolic Blood Pressure (mmHg)	131 ± 24.6	129 ± 18.5	0.969
Diastolic Blood Pressure (mmHg)	68.2 ± 13.1	66.1 ± 10.9	0.664
Heart Rate (bpm)	76.1 ± 16.6	77.1 ± 19.5	0.805
Comorbidities			
Chronic Kidney Disease, *n* (%)	40 (33.1%)	10 (35.7%)	0.826
Hypertension, *n* (%)	108 (89.0%)	20 (71.4%)	0.032
Type II Diabetes Mellitus, *n* (%)	45 (37.3%)	10 (35.7%)	>0.999
Dyslipidemia, *n* (%)	96 (79.7%)	23 (82.1%)	>0.999
Coronary Artery Disease, *n* (%)	76 (62.7%)	19 (67.9%)	0.668
Cardiac Function			
Left Ventricular Ejection Fraction (%)	56.5 ± 9.1	60.1 ± 6.2	0.017
Global Longitudinal Strain (%)	−12.66 ± 4.37	−14.95 ± 3.89	0.013

## Data Availability

The data are derived from patient health records and are not publicly available due to confidentiality. De-identified data may be requested from the corresponding author with IRB approval.
